# Mitochondrial oxidative DNA damage and exposure to particulate air pollution in mother-newborn pairs

**DOI:** 10.1186/s12940-016-0095-2

**Published:** 2016-01-20

**Authors:** Lotte Grevendonk, Bram G. Janssen, Charlotte Vanpoucke, Wouter Lefebvre, Mirjam Hoxha, Valentina Bollati, Tim S. Nawrot

**Affiliations:** EPIGET-Epidemiology, Epigenetics and Toxicology Lab-Department of Clinical Sciences and Community Health, Università degli Studi di Milano, Milan, Italy; Centre for Environmental Sciences, Hasselt University, Hasselt, Belgium; Belgian Interregional Environment Agency, Brussels, Belgium; Flemish Institute for Technological Research (VITO), Mol, Belgium; Department of Public Health & Primary Care, Occupational and Environmental Medicine, Leuven University, Leuven, Belgium

**Keywords:** 8-OHdG, Foetal development, Mitochondrial function, Oxidative damage, Particulate matter

## Abstract

**Background:**

Studies emphasize the importance of particulate matter (PM) in the formation of reactive oxygen species and inflammation. We hypothesized that PM exposure during different time windows in pregnancy influences mitochondrial 8-hydroxy-2′-deoxyguanosine (8-OHdG) levels, which is an established biomarker for oxidative stress, in both maternal and foetal blood.

**Methods:**

We investigated maternal (*n* = 224) and cord blood (*n* = 293) from mother-newborn pairs that were enrolled in the ENVIR*ON*AGE birth cohort. We determined mitochondrial 8-OHdG by quantitative polymerase chain reaction (qPCR). Multivariable regression models were used to assess the association between mitochondrial 8-OHdG with PM_10_ and PM_2.5_ exposure over various time windows during pregnancy_._

**Results:**

In multivariable analysis, PM_10_ exposure during the entire pregnancy was positively associated with levels of mitochondrial 8-OHdG in maternal blood. For an IQR increment in PM_10_ exposure an increase of 18.3 % (95 % confidence interval (CI): 5.6 to 33.4 %, *p* = 0.004) in 8-OHdG was observed. PM_10_ exposure during the last trimester of pregnancy was positively associated with levels of 8-OHdG (28.1, 95 % CI: 8.6 to 51.2 %, *p* = 0.004, for an IQR increment in PM_10_). In a similar way, PM_2.5_ exposure was significantly associated with an increase of mitochondrial 8-OHdG levels in maternal blood during the entire pregnancy (13.9, 95 % CI: 0.4 to 29.4 %, *p* = 0.04 for an IQR increment in PM_2.5_ exposure) and third trimester of pregnancy (28.1, 95 % CI: 3.6 to 58.4 %, *p* = 0.02 for an IQR increment in PM_2.5_ exposure). In umbilical cord blood, 8-OHdG levels were significantly associated with PM_10_ exposure during first and second trimester of pregnancy with respectively an increase of 23.0 % (95 % CI: 5.9 to 42.8 %, *p* = 0.007) and 16.6 % (95 % CI: 1.8 to 33.5 %, *p* = 0.03) for an IQR increment in PM_10_ exposure.

**Conclusions:**

We found PM-associated increased mitochondrial oxidative DNA damage during pregnancy in both mothers and their newborns. Accordingly, our study showed that particulate air pollution exposure in early life plays a role in increasing systemic oxidative stress, at the level of the mitochondria, both in mother and foetus.

**Electronic supplementary material:**

The online version of this article (doi:10.1186/s12940-016-0095-2) contains supplementary material, which is available to authorized users.

## Background

Particulate matter (PM) air pollution exposure is a known risk factor for the induction of inflammatory and oxidative stress responses [1–4], especially during the pregnancy period when increased levels of oxidative stress can be expected [5]. Mitochondria are the main intracellular source and target of reactive oxygen species (ROS) that are continually generated as by-products of mitochondrial respiration in the electron transport chain [6].

Among the different types of ROS-induced DNA damage, the oxidation of guanine and consequently the formation of 8-hydroxy-2′-deoxyguanosine (8-OHdG) is the most common one [7]. Oxidative DNA damage can be repaired by means of 8-OHdG removal from the nuclear, but also the mitochondrial genome, representing a steady-state level of oxidative stress within a cell [8]. Contrary to human nuclear DNA (nDNA), mitochondrial DNA (mtDNA) lacks the protective chromatin structure, histones, and introns. Moreover, the mtDNA repair mechanisms work less efficiently resulting in a 5 to 15 times higher mutation rate compared with nDNA [6, 9]. High and continuous exposure to PM-induced ROS in mitochondria are likely to cause oxidative mtDNA damage as reflected by increased amounts of mitochondrial 8-OHdG levels. Accumulation of mitochondrial 8-OHdG levels, and subsequent mtDNA mutations, are reported to be a risk factor for many diseases including diabetes [10] and cancer [11]. The strict maternal inheritance of mtDNA means that mtDNA mutations arising in the embryonic period can be dispersed throughout the body of the fetus. Several studies demonstrated an association between nuclear 8-OHdG levels and PM exposure [12–16] but whether exposure to PM during gestation influences mitochondrial 8-OHdG both in mothers and newborns is not known.

In the framework of the ENVIR*ON*AGE birth cohort (ENVIRonmental influence ON early AGEing), we hypothesized that mitochondrial oxidative DNA damage, as exemplified by mitochondrial 8-OHdG, is associated with airborne PM exposure during gestation.

## Methods

### Study population and data collection

We recruited mother-newborn pairs (only singletons) in the East-Limburg Hospital in Genk, Belgium who are part of the ongoing birth cohort “ENVIR*ON*AGE”. The current study comprises 293 cord blood samples collected immediately after delivery and 224 maternal blood samples collected at the maternal unit 24–48 h after delivery. The participation rate of eligible mothers in the birth cohort (mothers able to fill out a Dutch language questionnaire) was 61 % and enrolment was spread equally over all seasons of the year. Participating mothers had to complete a study questionnaire to provide detailed information on age, maternal education, occupation, ethnicity, smoking status, place of residence, pre-gestational body mass index (BMI), parity, and newborn’s ethnicity. Past-smokers were defined as those who had quit before pregnancy and smokers as having smoked before and during pregnancy. Women reported whether they were exposed to second-hand smoke at their home address. Maternal education was coded as low (no diploma or primary school), middle (high school), or high (college or university degree). We asked the mothers whether they consumed alcohol during pregnancy. Perinatal parameters such as newborn’s gender, birth date, birth weight and length, gestational age, Apgar score, and ultrasonographic data were collected after birth. The present study was conducted according to the principles outlined in the Helsinki Declaration (World Medical Association 2008) [17] for investigation of human subjects. Written informed consent was obtained from all participating mothers when they arrived at the hospital for delivery and was in accordance with procedures approved by the Ethical Committee of Hasselt University and East-Limburg Hospital in Genk.

### Exposure assessment

For each mother’s residential address, we interpolated the regional background levels of PM_2.5_ and PM_10_ (μg/m^3^) using a spatial temporal interpolation method (Krigin) [18] that uses pollution data collected in the official fixed site monitoring and land cover data obtained from satellite images (CORINE land cover data set) in combination with a dispersion model [19, 20]). This model chain provides daily PM_2.5_ and PM_10_ values using data both from the Belgian telemetric air quality network from point sources and line sources which are then interpolated to a high-resolution receptor grid. The date of conception was estimated based on ultrasound data. We explored potential critical windows of exposures during pregnancy using daily mean PM_2.5_ and PM_10_ concentrations averaged over various periods. Exposure windows of interest included each of the three trimesters of pregnancy, with trimesters being defined as: 1–13 weeks (first trimester), 14–26 weeks (second trimester), and 27 weeks to delivery (third trimester). The exposure during the entire pregnancy was calculated as the mean of all pregnancy days. Address changes during the period of pregnancy were taken into account when calculating the exposure windows (*n* = 16; 7.1 %).

### Sample collection and processing

Maternal and venous umbilical cord blood was collected after delivery in plastic BD Vacutainer® Plus Plastic K2EDTA Tubes (BD, Franklin Lakes, NJ, USA). Samples were centrifuged (3200 rpm for 15 min) to retrieve buffy coats and instantly frozen, first at −20 °C and later at −80 °C. Genomic DNA was isolated from buffy coat of maternal blood (*n* = 224) and umbilical cord blood (*n* = 293) using the QIAamp® DNA minikit (Qiagen, Inc., Venlo, the Netherlands) and stored at −80 °C until further use.

### Determination of the 8-OHdG levels in mitochondria

Mitochondrial 8-OHdG was measured using quantitative real-time polymerase chain reaction (qPCR) as previously described in detail [11, 21]. Briefly, 4 μl of DNA sample (6.25 ng/μl) was treated with 11 μl treatment mix containing RNase free water (8.7 μl/reaction), buffer NE 10× (1.5 μl/reaction), BSA 100× (0.15 μl/reaction), and the enzyme human oxoguanine glycosylase 1 (hOGG1) or RNase free water (0.625 μl/ reaction) for the treatment mix and non-treatment mix respectively. After an incubation of 1 h at 37 °C, samples were diluted 1:4 in RNase free water and a fragment of mtDNA (*MTF3212*/*R3319*) was amplified by qPCR using a master mix consisting of TaqMan® Fast Advanced Master Mix (Applied Biosystems; 5 μL/reaction), forward (0.5 μL/reaction) and reverse (0.5 μL/reaction) primers (F:5′-CACCCAAGAACAGGGTTTGT-3′ and R:5′-TTAACAACATACCCATGGCCA-3′), and 4 μl of treated or non-treated DNA. Samples were run in triplicate in a MicroAmp® Fast Optical 384-Well Reaction Plate compatible with the 7900HT Fast Real-Time PCR System (Applied Biosystems, Foster City, CA, USA). Each plate contained two non-template controls and four inter-run calibrators to account for plate variability. The thermal cycling profile started with 10 s at 95 °C, followed by 35 cycles of 15 s at 95 °C plus 1 min at 60 °C. qBase software (Biogazelle, Zwijnaarde, BE) was used to automatically average triplicate measurements that passed quality control and to correct for run-to-run differences [22]. Afterwards, differences in amplification efficiency between non-treated and treated DNA (∆Ct) were calculated.

### mtDNA content analysis

mtDNA content was measured using a qPCR assay by determining the ratio of two mitochondrial gene copy numbers (*MTF3212*/*R3319* and *MT*-*ND1*) to two single-copy nuclear control genes (*RPLP0* and *ACTB*) as previously described [17] but with small modification. Isolated genomic DNA (12.5 ng) was added to 7.5 μl mastermix consisting of Fast SYBR® Green I dye 2× (5 μl/reaction), forward and reverse primer (each 0.3 μl/reaction) and RNase free water (1.9 μl/reaction), for a final volume of 10 μl per reaction. Primer sequences (Additional file 1: Table S1) were diluted to a final concentration of 300 nM in the master mix. Samples were run in triplicate in 384-well format. Real-time PCR was performed using the 7900HT Fast Real-Time PCR System (Applied Biosystems, Foster City, CA, USA) with following thermal cycling profile: 20 s at 95 °C, followed by 40 cycles of 1 s at 95 °C and 20 s at 60 °C, ending with melting curve analysis (15 s at 95 °C, 15 s at 60 °C, 15 s at 95 °C). qBase software (Biogazelle, Zwijnaarde, BE) was used to normalize data and correct for run-to-run differences [22].

### Statistical analysis

For database management and statistical analysis, we used the SAS software program (version 9.2; SAS Institute Inc., Cary, NC, USA). mtDNA 8-OHdG levels and mtDNA content data were log_10_-transformed to improve normality. Pearson correlation coefficients and multiple linear regression were used to assess the correlation and association between PM_2.5_ or PM_10_ exposure with mtDNA 8-OHdG in maternal blood and cord blood and to assess the correlation and association between mtDNA 8-OHdG and mtDNA content in maternal and cord blood. The models for maternal blood were controlled for a priori chosen variables including maternal age, gestational age, smoking status, maternal education, alcohol consumption during pregnancy, and season at conception, while the models for cord blood additionally were adjusted for gender and date of delivery. β regression coefficients represent a relative percentage change for an increment between the interquartile range (IQR; 25th–75th percentile) in the independent variable.

In a sensitivity analysis, we assessed the association between PM_2.5_ or PM_10_ exposure during pregnancy and 8-OHdG levels in maternal and cord blood while excluding women who smoked during pregnancy. We explored whether assisted reproductive technologies may alter the association between PM exposure and cord or maternal 8-OHdG levels. Lastly, we investigated whether PM exposure or 8-OHdG levels were associated with pregnancy outcomes such as birth weight using linear regression models and small for gestational age using logistic regression models. The Shapiro-Wilk statistic and Q-Q plots of the residuals were used to test the assumptions of all linear models.

## Results

Lifestyle factors were collected by means of a self-reported questionnaire and are displayed in Table 1. In this study, 293 pregnant women were included with an average (SD) age of 29 (4.8) years (range: 17–44 years). Mean pre-gestational BMI of participating mothers averaged 24.2 (4.7) kg/m^2^. Most women (*n* = 195, 66.5 %) never smoked cigarettes whereas 47 mothers (16.1 %) reported to have smoked during pregnancy and 51 women (17.4 %) reported to have smoked before pregnancy. Most mothers (*n* = 235, 80.2 %) did not consume alcohol during their pregnancy, while 19.8 % (*n* = 58) reported to have consumed alcohol occasionally. Most women (90.4 %) had a spontaneous pregnancy without the use of artificial reproductive technology. The newborns, among them 140 males (47.8 %), had a mean gestational age of 39.2 (1.2) weeks. Table 2 displays the daily outdoor PM_10_ and PM_2.5_ exposure levels averaged for the entire pregnancy and each of the three trimesters of pregnancy. Median (25th–75th percentile) trimester-specific PM_10_ exposure was 19.2 (16.6–23.8) μg/m^3^ for the first trimester, 21 (17.4–23.8) μg/m^3^ for the second trimester, 22.2 (17.3–26.1) μg/m^3^ for the third trimester, and 21.4 (19.8–22.8) μg/m^3^ for the entire pregnancy.

**Table 1:** Characteristics of mother-newborn pairs (*n* = 293)

Maternal characteristics	Mean ± SD or range and number (%)
Age, year	29 ± 4.8
Pre-gestational BMI, kg/m^2^	24.2 ± 4.7
Gestational age, weeks	39.2 ± 1.2
Maternal education	
Low	39 (13.3)
Middle	98 (33.5)
High	156 (53.2)
Smoking	
Never-smoker	195 (66.5)
Past-smoker	51 (17.4)
Smoker	47 (16.1)
Second-hand smoke exposure^a^	
Not exposed	260
Non-smoker, exposed	14
Smoker, exposed	10
Alcohol	
No	235 (80.2)
Occasionally	58 (19.8)
Assisted reproductive technology	
Spontaneous	265 (90.4)
Hormonal	7 (2.4)
In vitro fertilization	12 (4.1)
Intracytoplasmic sperm injection	4 (1.4)
Artificial insemination	5 (1.7)
Newborn’s gender	
Male	140 (47.8)
Female	153 (52.2)
Season at delivery	
Fall	73 (24.9)
Winter	67 (22.9)
Spring	94 (32.1)
Summer	59 (20.1)

^a^Data available for 284 individuals

**Table 2:** Exposure characteristics during pregnancy (*n* = 293)

Variable	Min	25 % (Q1)	Median	75 %(Q3)	Max	IQR
PM_10_, μg/m^3^						
1st trimester	12.3	16.6	19.2	23.8	37.6	7.2
2nd trimester	11.1	17.4	21.0	23.8	34.6	6.4
3rd trimester	11.5	17.3	22.2	26.1	37.3	8.8
Entire pregnancy	15.3	19.8	21.4	22.8	27.3	3.0
PM_2.5_, μg/m^3^						
1st trimester	7.8	11.8	14.3	19.9	29.6	8.1
2nd trimester	7.9	12.1	16.2	19.9	28.6	7.8
3rd trimester	7.9	11.9	17.1	22.0	31.4	10.1
Entire pregnancy	11.3	15.1	16.6	18.0	22.0	2.9

Mitochondrial 8-OHdG levels in maternal blood were positively correlated with PM_10_ and PM_2.5_ exposure during the entire pregnancy. An IQR increment in PM_10_ exposure was associated with an increase in maternal mitochondrial 8-OHdG levels of 15.7 % (95 % confidence interval (CI): 4.8 to 27.6 %, *p* = 0.004). For an IQR increment in PM_2.5_ exposure a positive trend in 8-OHdG levels was observed (10.1 %, 95 % CI: −0.2 to 21.3 %, *p* = 0.05). The association remained significant after adjustment for maternal age, gestational age, smoking status, maternal education, alcohol consumption and season at conception (Table 3). An IQR increment in PM_10_ and PM_2.5_ during the entire pregnancy was associated with a change in maternal 8-OHdG of +18.3 % (95 % CI: 5.6 to 32.4 %, *p* = 0.004) and +13.9 % (95 % CI: 0.4 to 29.4 %, *p* = 0.04) respectively. Considering the exposures during the trimesters separately, a positive association was observed between 8-OHdG levels in maternal blood with PM_10_ and PM_2.5_ exposure during the third trimester of pregnancy (+28.1 %, 95 % CI: 8.6 to 51.2 %, *p* = 0.004 and +28.1 %, 95 % CI: 3.6 to 58.4 %, *p* = 0.02 for an IQR increment in PM_10_ and PM_2.5_ exposure respectively). Exposure to PM_10_ and PM_2.5_ during the first and second trimester of pregnancy was not significantly associated with maternal 8-OHdG levels, although a positive trend was observed (*p* > 0.14).

**Table 3:** Estimated change of mitochondrial 8-OHdG in maternal blood associated with PM_10_ and PM_2.5_ exposure during pregnancy (*n* = 224)

	PM_10_^a^			PM_2.5_^a^		
Time window	Percent change^b^	95 % CI	*p*-Value	Percent change^b^	95 % CI	*p*-Value
Trimester 1 (1–13w)	7.3	−7.1, 23.9	0.34	3.0	−13.6, 22.7	0.74
Trimester 2 (14–26w)	12.4	−3.89, 31.5	0.14	13.0	−9.1, 40.4	0.27
Trimester3 (27w-delivery)	28.1	8.6, 51.2	0.004	28.1	3.6, 58.4	0.02
Entire pregnancy	18.3	5.6, 32.4	0.004	13.9	0.4, 29.4	0.04

^a^The model is adjusted for maternal age, gestational age, smoking status, maternal education, alcohol consumption during pregnancy, and season at conception.

^b^The effect size is calculated as a relative percent change for an IQR increment in PM_10_ or PM_2.5_ exposure (μg/m^3^) at the mother’s residence during the different time windows. The IQR for the different time windows for PM_10_ and PM_2.5_ is given in Table 2

To investigate whether the effects we observed in maternal blood are passed through or translated to the foetus, we determined levels of 8-OHdG in cord blood of 293 newborns. After adjustment of the aforementioned variables plus newborns gender and delivery date, we did not observe a significant association between cord blood 8-OHdG levels and PM_10_ or PM_2.5_ exposure during the entire pregnancy although a positive trend was observed (*p* > 0.11). However, considering the separate exposure windows, we observed a significant positive association between cord blood 8-OHdG and PM_10_ exposure during the first and second trimester of pregnancy (Table 4). An IQR increment in PM_10_ exposure was associated with a change in mitochondrial 8-OHdG levels of +23.0 % (95 % CI: 5.9 to 42.8 %, *p* = 0.007 and +16.6 % (95 % CI: 1.8 to 33.5 %, *p* = 0.03) respectively. Exposure to PM_2.5_ during the first and second trimester of pregnancy was not significantly associated although a positive trend was shown (+15.8, 95 % CI: −2.4 to 37.6 %, *p* = 0.09 and +12.1, 95 % CI: −5.7 to 33.1 %, *p* = 0.19). Contrary, exposure to PM_10_ and PM_2.5_ during the third trimester showed an inverse trend with 8-OHdG levels in cord blood, although this was not significant.

**Table 4:** Estimated change of mitochondrial 8-OHdG in cord blood associated with PM_10_ and PM_2.5_ exposure during pregnancy (*n* = 293)

	PM_10_^a^			PM_2.5_^a^		
Time window	Percent change^b^	95 % CI	*p*-Value	Percent change^b^	95 % CI	*p*-Value
Trimester 1 (1–13w)	23.0	5.9, 42.8	0.007	15.8	−2.4, 37.6	0.09
Trimester 2 (14–26w)	16.6	1.8, 33.5	0.03	12.1	−5.7, 33.1	0.19
Trimester 3 (27w-delivery)	−13.4	−26.2, 1.7	0.08	−14.9	−29.4, 2.6	0.09
Entire pregnancy	8.7	−2.0, 20.6	0.11	3.1	−7.0, 14.4	0.56

^a^The model is adjusted for maternal age, gestational age, smoking status, maternal education, alcohol consumption during pregnancy, season at conception, gender, and date of delivery.

^b^The effect size is calculated as a relative percent change for an IQR increment in PM_10_ or PM_2.5_ exposure (μg/m^3^) at the mother’s residence during the different time windows. The IQR for the different time windows for PM_10_ and PM_2.5_ is given in Table 2

To explore the functional significance of the association between mitochondrial 8-OHdG with exposure to PM, we evaluated the association between mtDNA content, a measure of altered mitochondrial function, and 8-OHdG levels in maternal and cord blood. Pregnant mothers with high levels of 8-OHdG exhibited high levels of mtDNA content in their blood (r = 0.44, *p* < 0.0001). Likewise, we observed a strong positive correlation between mitochondrial 8-OHdG and mtDNA content in cord blood (r = 0.58, *p* < 0.0001). These associations of 8-OHdG and mtDNA content in cord blood and maternal blood remained significant after adjustment for the same covariates as in the maternal and cord blood model (β = 0.38, 95 % CI: 0.28 to 0.49, *p* < 0.0001 and β = 0.52, 95 % CI: 0.43 to 0.61, *p* < 0.0001 respectively).

In a sensitivity analysis, we repeated all statistical analyses while excluding women who smoked during pregnancy (*n* = 47). This did not alter the reported associations between PM exposure and 8-OHdG in maternal and cord blood except that the associations between PM_2.5_ exposure and maternal 8-OHdG attenuated and did not reach significance anymore (Additional file 1: Table S2 and Table S3). Secondly, adjusting our main models with assisted reproductive technology did not change our findings (results not shown). Lastly, neither PM exposure nor 8-OHdG levels in cord and maternal blood were associated with birth weight or small for gestational age (Additional file 1: Table S4).

## Discussion

Pregnancy is a state of increased physiological oxidative stress [5], and maternal exposure to particulate air pollution might exacerbate oxidative damage [1]. Our key finding was that maternal particulate air pollution exposure was positively associated with mitochondrial 8-OHdG, a marker of oxidative DNA damage, in maternal and cord blood. Accordingly, our research showed that particulate air pollution in early life plays a role in increasing systemic oxidative stress, at the level of the mitochondria, both in mother and foetus.

Most studies to date have analyzed excreted levels of 8-OHdG in urine, reflecting nuclear DNA damage, instead of mitochondrial 8-OHdG levels like in our study. Consistent with our results, most of these studies observed a positive association between nuclear 8-OHdG levels in urine and PM exposure [14–16, 23–25] or tobacco smoke exposure [16, 26–29]. Also in human and in vitro blood lymphocytes, increases in nuclear 8-OHdG levels have been associated with PM exposure [12, 13]. To the best of our knowledge this is the first study investigating 8-OHdG levels, specifically in the mitochondrial genome, in association with prenatal PM exposure. The advantage of measuring 8-OHdG in mtDNA compared with the excreted form of 8-OHdG is that this method gives a more direct measurement of oxidative damage occurring within mitochondria, and therefore, reflecting mitochondrial function. Repair of 8-OHdG lesions from the mitochondrial genome ensures a steady-state level of oxidative stress within the mitochondrion [8]. However, during long periods of PM-induced ROS exposure, 8-OHdG levels may accumulate in the body or tissue of both the mother and foetus and, therefore, oxidative mtDNA damage may be more extensive and persistent compared to nuclear DNA damage [30]. This accumulation over a long period of time could explain the association between 8-OHdG levels and PM_10_ exposure over the entire pregnancy. The strict maternal inheritance of mtDNA could imply that increased oxidative stress levels and mtDNA mutations present within mitochondria of the mother, predefine oxidative mtDNA damage levels within the foetus. Our findings showed that the association between maternal particulate air pollution and newborn mtDNA damage was most significantly associated with first and second trimester exposure windows, pointing to predefined mtDNA oxidative damage in susceptible windows of exposure during pregnancy. In contrast, the inverse trend we observed in trimester 3 could mean that the foetus is somewhat protected from maternal-derived ROS or might be the result of an early decrease in the number of mitochondria due to mitophagy [31] with less endogenous ROS formation. Early and mid-pregnancy exposure windows are critical for adverse newborn health outcomes, including preterm birth [32] and increased risk of gestational diabetes mellitus [33]. Unfortunately, we did find an association between gestational PM exposure and adverse pregnancy outcomes such as birth weight or small for gestational age.

However, our findings showed that mtDNA content, a measure of altered mitochondrial function, was associated with 8-OHdG levels in both maternal and cord blood [11, 34]. Exposure to PM-induced ROS is likely to cause oxidative mtDNA damage and subsequent mutations of mtDNA, leading to mitochondrial heteroplasmy or mixed mitochondrial populations [9, 35]. If accumulation of 8-OHdG and somatic mtDNA mutations exceed a critical threshold, they can cause a biochemical defect in the mitochondrial respiratory chain and could result in a progressive decline of mitochondrial function [10, 34, 36]. Previously, we have shown that PM_2.5_ exposure during pregnancy affects global DNA methylation levels in placental tissue [37], and we found subtle differences in methylation levels of specific regions in the mitochondrial genome [31]. Besides metabolic disorders, the stochastic accumulation of mtDNA mutations encompasses the pathophysiological basis of disease processes including aging, neurodegenerative diseases, and cancer [38].

Oxidative modifications in mitochondria represent a potential link between adverse insults and altered foetal development. Increased oxidative stress levels in pregnancy, including levels of 8-OHdG, have been implicated in pregnancy complications and adverse outcomes such as gestational diabetes mellitus [39] and preeclampsia [40]. In our study, we did not observe an association between pregnancy outcomes such as birth weight or small for gestational age and 8-OHdG levels in maternal or cord blood. Nevertheless, the mitochondrial oxidative DNA damage-mediated health effects of PM exposure need to be further elucidated.

The present study must be interpreted within the context of its limitations and strengths. We measured 8-OHdG in mtDNA in mothers at the end of their pregnancy which consequently impairs the ability to investigate changes in 8-OHdG in mitochondrial DNA during the entire pregnancy. However, a strength is that mitochondria capture long-term oxidative stress accumulation, and having samples of mother-newborn pairs, we were able to explore both effects on the mother and foetus. Although our results were consistent after multiple adjustments, we cannot exclude the possibility of residual confounding by some unknown factor that is associated with both 8-OHdG levels and ambient air pollution. Our findings were not influenced by other factors that possibly could alter the association between PM exposure and 8-OHdG levels or mtDNA damage such as smoking and use of assisted reproductive technology.

Prenatal exposure to PM_10_ in Flanders (Belgium), which is on average 21.4 μg/m^3^ in the mother-newborns pairs of our cohort, is associated with elevated mitochondrial oxidative stress, reflected by increased 8-OHdG levels in the mother and foetus. Our findings are generalisable as our study population is representative for the gestational segment of the population at large. Although our concentrations might be relatively high for affluent Western societies, the levels are low compared to other parts of the world e.g. in Lahore (Pakistan) with average PM_10_ concentrations above 700 μg/m^3^ and average PM_2.5_ concentrations above 180 μg/m^3^ [41].

## Conclusions

We demonstrated that mitochondrial oxidative DNA damage in both newborns and their mothers was associated with particulate air pollution exposure during pregnancy. Future molecular epidemiological studies in newborns are required to clarify the impact of mitochondrial oxidative DNA damage on newborn’s and children’s health.

## Additional file

Additional file 1: Table S1. Mitochondrial and nuclear primer sequence information based upon Assembly GRCh37/hg19 of the UCSC genome browser for mtDNA content measurement. **Table S2.** Estimated change of mitochondrial 8-OHdG in maternal blood associated with PM_10_ and PM_2.5_ exposure during pregnancy while excluding women who continued smoking during pregnancy (*n* = 193). **Table S3.** Estimated change of mitochondrial 8-OHdG in cord blood associated with PM_10_ and PM_2.5_ exposure during pregnancy while excluding women who continued smoking during pregnancy (*n* = 246). **Table S4.** PM exposure, 8-OHdG levels and pregnancy outcomes. (DOCX 24 kb)

## Abbreviations

8-OHdG: 8-hydroxy-2′-deoxyguanosine
CI: confidence interval
Ct: threshold cycles
hOGG1: human oxoguanine glycosylase 1
mtDNA: mitochondrial DNA
nDNA: nuclear DNA
PM: particulate matter
PM_2.5_: particles with a diameter smaller than 2.5 μm
PM_10_: particles with a diameter smaller than 10 μm
qPCR: quantitative real-time polymerase chain reaction
ROS: reactive oxygen species
