# Supplementary material for: Mitochondrial oxidative DNA damage and exposure to particulate air pollution in mother-newborn pairs
Source: Environ Health. 2016 Jan 20;15:10. doi: 10.1186/s12940-016-0095-2 (PMC4719654; doi:10.1186/s12940-016-0095-2)
Supplement: Additional file 1: Table S1. — Mitochondrial and nuclear primer sequence information based upon Assembly GRCh37/hg19 of the UCSC genome browser for mtDNA content measurement. Table S2. Estimated change of mitochondrial 8-OHdG in maternal blood associated with PM10 and PM2.5 exposure during pregnancy while excluding women who continued smoking during pregnancy (n = 193). Table S3. Estimated change of mitochondrial 8-OHdG in cord blood associated with PM10 and PM2.5 exposure during pregnancy while excluding women who continued smoking during pregnancy (n = 246). Table S4. PM exposure, 8-OHdG levels and pregnancy outcomes. (DOCX 24 kb) [file 12940_2016_95_MOESM1_ESM.docx]

# Supplementary material

# Mitochondrial oxidative DNA damage and exposure to particulate air pollution in mother and newborns

**Lotte Grevendonk^1^, Bram G. Janssen^2^, Charlotte Vanpoucke^3^, Wouter Lefebvre^4^, Mirjam Hoxha^1^, Valentina Bollati^1^, Tim S. Nawrot^2,5^**

# Table S1. Mitochondrial and nuclear primer sequence information based upon Assembly GRCh37/hg19 of the UCSC genome browser for mtDNA content measurement.

| **Gene symbol** | **Chr** | **Amplicon**  **start-end** | **Primer set** | **Amplicon (bp)** |
| --- | --- | --- | --- | --- |
| *MTF3212/R3319* | M | 3213- | F:5’-CACCCAAGAACAGGGTTTGT-3’ | 108 |
|  |  | 3320 | R:5’-TTAACAACATACCCATGGCCA-3’ |  |
| *MT-ND1* | M | 3314- | F:5’-ATGGCCAACCTCCTACTCCT-3’ | 115 |
|  |  | 3428 | R:5’-AAAGGCCCCAACGTTGTAG-3’ |  |
| *RPLP0* | 12 | 120636904- | F:5’-CCCAATTGTCCCCTTACCT-3’ | 85 |
|  |  | 120636988 | R:5’-GAACACAAAGCCCACATTCC-3’ |  |
| *ACTB* | 7 | 5567833- | F:5’-ACTCTTCCAGCCTTCCTTCC-3’ | 102 |
|  |  | 5567934 | R:5’-TGTGGAAGCTAAGTCCTGCC-3’ |  |
| Mitochondrial forward primer from nucleotide 3212 and reverse primer from nucleotide 3319 (*MTF3212/R3319*); Mitochondrial encoded NADH dehydrogenase 1 (*MT-ND1*); Acidic ribosomal phosphoprotein P0 (*RPLP0*); Beta actin (*ACTB*). | | | | |

**Table S2: Estimated change of mitochondrial 8-OHdG in maternal blood associated with PM_10_ and PM_2.5_ exposure during pregnancy while excluding women who continued smoking during pregnancy (*n* = 193).**

|  | **PM_10_^b^** | | |  | **PM_2.5_^b^** | | |
| --- | --- | --- | --- | --- | --- | --- | --- |
| **Time window** | **Percent change^a^** | **95% CI** | ***p*-Value** |  | **Percent change^a^** | **95% CI** | ***p*-Value** |
| Trimester 1 (1-13w) | 6.6 | -11.8, 21.5 | 0.67 |  | 8.3 | -18.2, 20.7 | 0.95 |
| Trimester 2 (14-26w) | 7.3 | -6.0, 31.6 | 0.21 |  | 9.4 | -11.5, 41.1 | 035 |
| Trimester3 (27w-delivery) | 8.4 | 1.2, 45.9 | 0.04 |  | 10.0 | -5.5, 49.3 | 0.14 |
| Entire pregnancy | 3.2 | 0.3, 29.3 | 0.04 |  | 3.3 | -5.3, 25.1 | 0.23 |

^a^ The effect size is calculated as a relative percent change for an IQR increment in PM_10_ or PM_2.5_ exposure (µg/m^3^) at mother’s residence during the different time windows. IQR for the different time windows for PM_10_ and PM_2.5_ is given in Table 2.

^b^ The model is adjusted for maternal age, gestational age, smoking status, maternal education, alcohol consumption during pregnancy, and season at conception.

Table S3: Estimated change of mitochondrial 8-OHdG in cord blood associated with PM_10_ and PM_2.5_ exposure during pregnancy while excluding women who continued smoking during pregnancy (*n* = 246).

|  | **PM_10_^b^** | | |  | **PM_2.5_^b^** | | |
| --- | --- | --- | --- | --- | --- | --- | --- |
| **Time window** | **Percent change^a^** | **95% CI** | ***p*-Value** |  | **Percent change^a^** | **95% CI** | ***p*-Value** |
| Trimester 1 (1-13w) | 7.3 | 4.7, 44.4 | 0.01 |  | 8.1 | -3.0, 39.9 | 0.10 |
| Trimester 2 (14-26w) | 6.4 | 1.5, 34.5 | 0.03 |  | 7.8 | -6.6,34.3 | 0.22 |
| Trimester 3 (27w-delivery) | 8.8 | -26.0, 3.9 | 0.12 |  | 10.1 | -29.9, 3.5 | 0.11 |
| Entire pregnancy | 3.0 | -2.12, 22.5 | 0.11 |  | 2.9 | -7.8, 14.9 | 0.61 |

^a^ The effect size is calculated as a relative percent change for an IQR increment in PM_10_ or PM_2.5_ exposure (µg/m^3^) at mother’s residence during the different time windows. IQR for the different time windows for PM_10_ and PM_2.5_ is given in Table 2.
^b^ The model is adjusted for maternal age, gestational age, smoking status, maternal education, alcohol consumption during pregnancy, season at conception, gender, and date of delivery.

Table S4: PM exposure, 8-OHdG levels and pregnancy outcomes.

|  | **PM_10_** | |  | **PM_2.5_** | |  | **Maternal 8-OHdG** | |  | **Cord 8-OHdG** | |
| --- | --- | --- | --- | --- | --- | --- | --- | --- | --- | --- | --- |
| **Variable** | **β*^*^*** | **95% CI** |  | **β*^*^*** | **95% CI** |  | **β*^*^*** | **95% CI** |  | **β*^*^*** | **95% CI** |
| Birth weight | 27.6 | -32.2, 87.4 |  | 38.2 | -18.8, 98.4 |  | 16.2 | -44.0, 76.4 |  | -30.4 | -82.4, 21.6 |
| **Variable** | **OR** | **95% CI** |  | **OR** | **95% CI** |  | **OR** | **95% CI** |  | **OR** | **95% CI** |
| SGA | 1.54 | 0.83, 2.86 |  | 1.30 | 0.73, 2.34 |  | 0.96 | 0.50, 1.84 |  | 0.69 | 0.43, 1.11 |

OR = odds ratio. SGA = small for gestational age (infants born with a birth weight less than the 10^th^ percentile).

^*^ β represents a change in grams for an IQR increment in PM exposure or a doubling in 8-OHdG levels in maternal and cord blood.

The linear regression and logistic models are adjusted for gender and gestational age.
